# Associations of Living Environment Attributes with Attitudes Towards Aging Among Older Adults in Shanghai China

**DOI:** 10.1093/geroni/igaa057.2973

**Published:** 2020-12-16

**Authors:** Minzhi Ye, Lin Chen

**Affiliations:** 1 Benjamin Rose Institute on Aging, Cleveland, Ohio, United States; 2 Fudan University, Shanghai, China

## Abstract

This study examined the relationships between several attributes of living environment and attitudes towards aging among persons of age 75 years and older in Shanghai, China. The data were collected using self-administered surveys in a study of successful aging in 2018. Respondents included 139 persons of age 75 years and older living in 12 neighborhoods in Shanghai. Attitudes towards aging were measured by indices of happiness, time use and worry about the future. Using BaiduMap and GIS ArcPro, we quantified accessibility to neighborhood living resources as numbers of recreational facilities, medical facilities, parks, and grocery stores located within 500 meters of each respondent’s home. The analysis found that more positive attitudes towards aging were correlated with less depressive symptom as measured by CES-D and greater numbers of neighborhood resources near home, especially greater number of grocery stores near home. In conclusion, accessibility to neighborhood resources may influence attitudes towards aging.

